# Geriatric Total Hip Arthroplasty: Fixation Strategies, Approaches and Outcomes

**DOI:** 10.26502/josm.511500255

**Published:** 2026-03-12

**Authors:** Bahram Saber, Devendra K. Agrawal

**Affiliations:** Department of Translational Research, College of Osteopathic Medicine of the Pacific, Western University of Health Sciences, Pomona CA 91766, USA

**Keywords:** Arthroplasty, Arthroplasty survivorship, Cemented vs. cementless fixation, Direct Anterior Approach (DAA), Geriatric Orthopedics, Total Hip Arthroplasty (THA), Osteoporosis, Perioperative Outcomes, Periprosthetic Joint Infection (PJI), Surgical Biomechanics

## Abstract

Total hip arthroplasty (THA) is a cornerstone of geriatric medicine, yet the selection of optimal fixation strategies and surgical approaches remains a subject of intense clinical debate. As global demographics shift toward an aging population, the demand for THA is projected to rise exponentially, necessitating a thorough evaluation of perioperative outcomes and long-term survivorship. This review critically analyzes recent 2024–2025 evidence regarding the biomechanical stability and clinical efficacy of cemented versus cementless fixation in patients aged 70 years or older. Current literature suggests that while cementless technology dominates younger cohorts, cemented fixation provides superior initial rotational stability in the osteoporotic environment, significantly reducing the risk of intraoperative periprosthetic fractures. Furthermore, this report examines the impact of the Direct Anterior Approach (DAA) compared to the Posterior Approach (PA) within the framework of Enhanced Recovery After Surgery (ERAS), highlighting the DAA’s benefits in minimizing soft-tissue trauma and reducing hospital length of stay. Finally, we synthesize modifiable and non-modifiable risk factors for periprosthetic joint infection (PJI) and fracture progression. Understanding these multifaceted factors is essential for tailoring personalized surgical interventions and improving functional recovery in the geriatric population.

## 1. Introduction

Total hip arthroplasty (THA) remains one of the most successful interventions in modern medicine, providing a definitive solution for end-stage hip osteoarthritis and femoral neck fractures, with more than 95% of implants surviving beyond 10 years [1]. As the global population ages, the demand for geriatric hip reconstruction is rising exponentially, with annual procedures projected to increase by 284% in the United States by 2040 compared to 2014 [2]. This surge necessitates refined strategies that prioritize immediate mechanical stability, accelerated functional recovery, and the mitigation of devastating postoperative complications [3]. The choice between cemented and cementless femoral stem fixation in elderly patients remains a central controversy in contemporary orthopedic practice [4]. Historically, cemented THA utilizing polymethylmethacrylate (PMMA) was the gold standard due to its immediate stability regardless of bone quality [4]. In contrast, modern cementless technology relies on biological osseointegration through press-fit fixation and is widely used in contemporary practice [4]. The application of cementless fixation in the elderly is complicated by the presence of osteoporosis and concerns regarding aseptic loosening, which remains one of the most common causes of THA failure [3,5]. This report provides a critical analysis of current evidence regarding fixation strategies, surgical approaches, and the evolving understanding of complication risk factors in the geriatric population.

## 2. Biomechanical Foundations of Geriatric Fixation

Fixation in the osteoporotic geriatric femur must withstand substantial rotational forces to prevent early failure.

## 3. Rotational Stability and Torque Resistance

**Figure 1:**
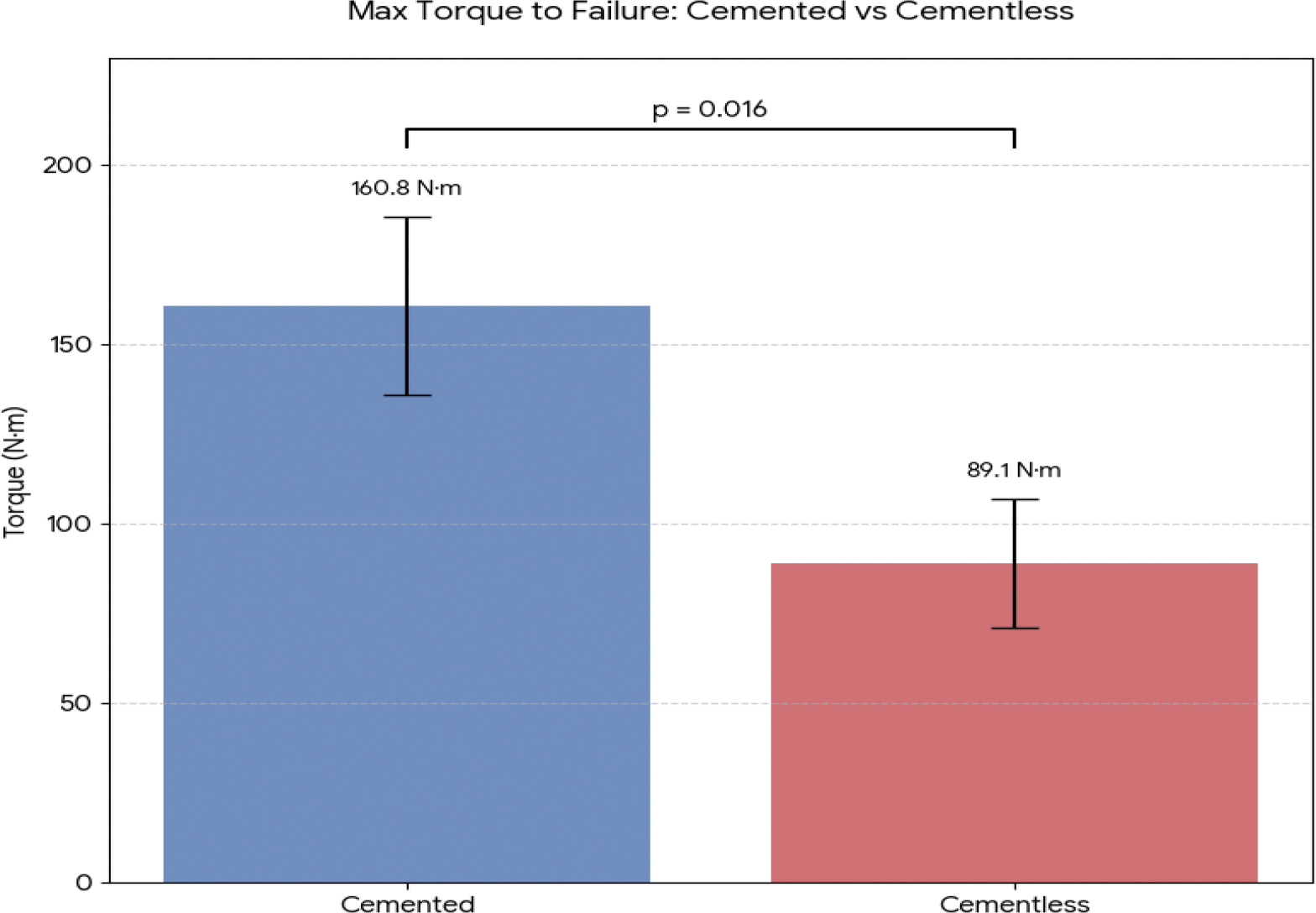
Comparison of Maximum Torque to Failure Between Cemented and Cementless Stems in an Intraoperative Periprosthetic Femur Fracture Model (N=6). Values are presented as mean ± SD. Data are compiled from the published findings of Watanabe et al. [6]. The level of significant difference in the maximum torque to failure between the two fixation methods is shown with the p value.

Recent 2025 biomechanical models of Vancouver A2-type periprosthetic fractures have utilized fourth-generation composite femurs to simulate the geriatric bone environment. Cemented stems demonstrated markedly superior stability compared to cementless designs, with a maximum torque to failure of 160.8 ± 24.9 N·m for cemented stems versus 89.1 ± 18.0 N·m for cementless stems (p = 0.016) [6]. Figure 1 illustrates this difference in rotational strength between fixation methods. This superior stability is attributed to the “load-dispersing” effect of the cement mantle, which distributes stress across the bone–implant interface and reduces localized pressure on the fracture line [6]. Strain gauge measurements indicate that peak strain at the medial fracture line is significantly lower in cemented constructs (p = 0.011) [6], further supporting the biomechanical advantage of cementation in osteoporotic bone.

## 4. Perioperative Outcomes and Recovery Trajectories

The choice of fixation directly influences the immediate postoperative period, hospital utilization, and discharge safety in elderly cohorts [7].

## 5. Hospital Length of Stay (LOS) and Discharge

**Figure 2:**
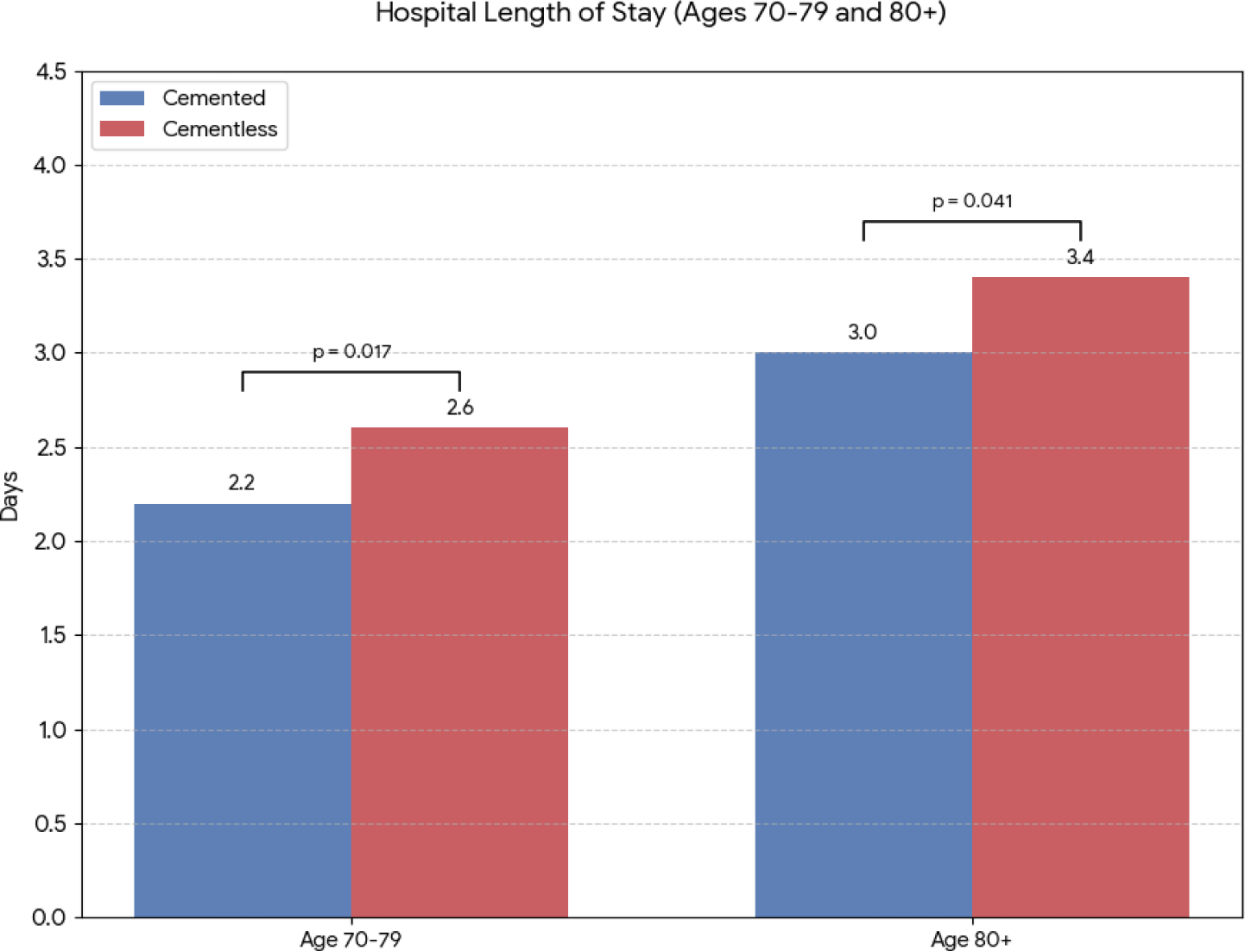
Comparison of Hospital Length of Stay (LOS) Between Cemented and Cementless THA in Geriatric Patients, (N≈2,100). Values are presented as mean days. Data are compiled from the published findings of Haider et al. [7]. The level of significant difference in hospital LOS between cemented and cementless stems in the 70–79 years group and the ≥80 years group is shown with the p values.

**Figure 3:**
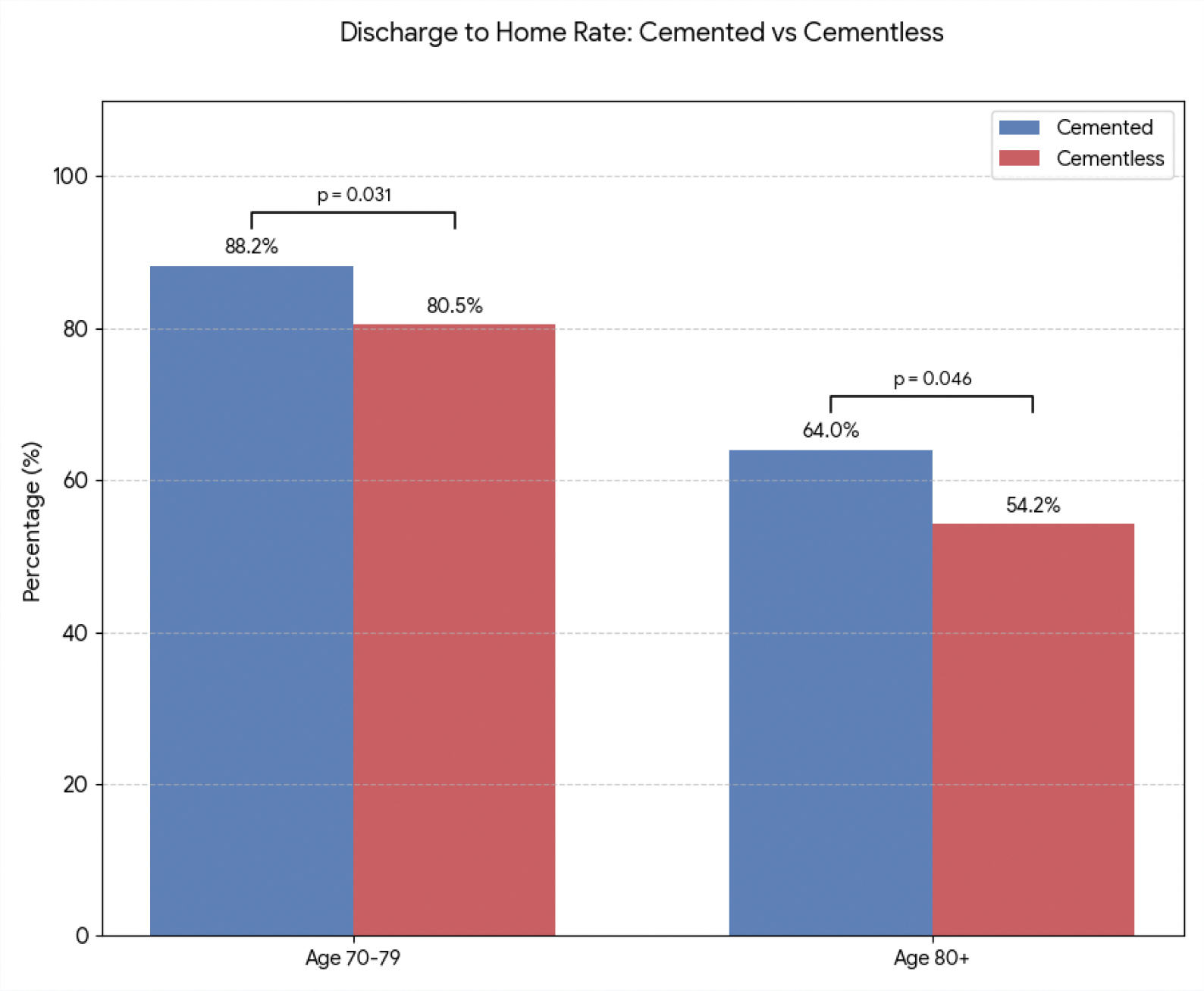
Rates of Discharge to Home for Cemented Versus Cementless THA in Geriatric Patients, (N≈2,100). Values are presented as percentages of patients meeting criteria for safe home discharge. Data are compiled from the published findings of Haider et al. [7]. The level of significant difference in home discharge rates between cemented and cementless stems in the 70–79 years group and the ≥80 years group is shown with the p values.

Propensity-matched analyses of over 2,000 patients have revealed that while cemented THA often involves slightly longer operative times due to cement preparation and curing, it leads to significantly shorter hospital stays in the elderly [7]. Patients aged ≥70 undergoing cemented THA demonstrate reduced LOS and higher rates of home discharge compared to matched cementless cohorts [7]. This is evident in Figure 2 and Figure 3, which summarize large-cohort findings for postoperative LOS and discharge disposition. Notably, the use of cement in osteoporotic patients confers immediate fixation stability, allowing earlier weight-bearing and mobilization, which likely contributes to these favorable perioperative outcomes.

## 6. Surgical Approach: Direct Anterior (DAA) vs. Posterior (PA)

A significant trend in 2025 is the evaluation of surgical approach within ERAS protocols and its impact on patient outcomes. The DAA has gained popularity for its tissue-sparing technique, especially in geriatric patients [8].

## 7. Early Functional Advantages of DAA

**Figure 4:**
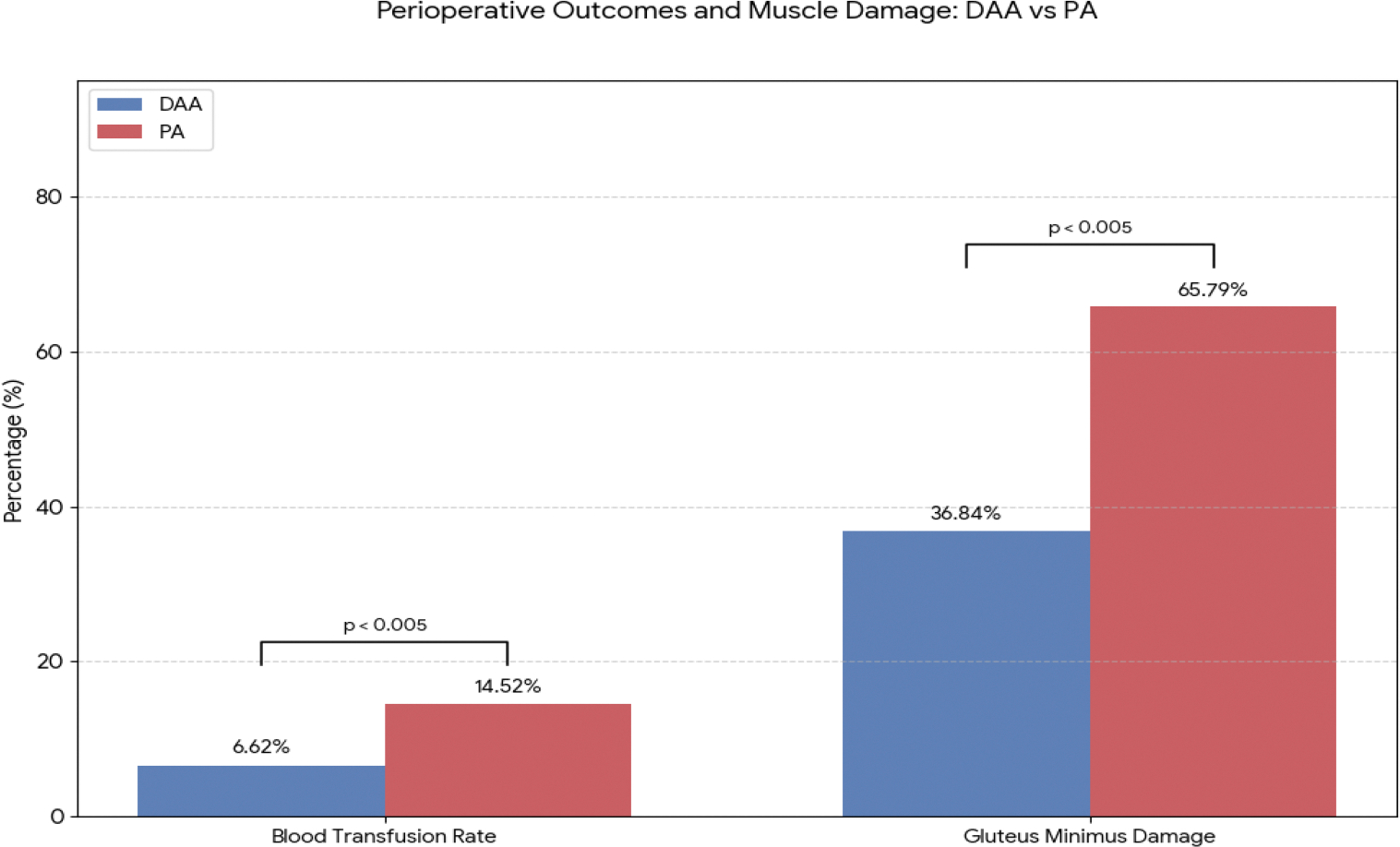
Key Perioperative and Anatomical Outcome Differences Between the Direct Anterior Approach (DAA) and Posterior Approach (PA), (N=46,367). Values are presented as percentages of patients. Data are compiled from the published findings of Xu et al. [8]. The level of significant difference in blood transfusion rates and gluteus minimus muscle damage between the DAA and PA is shown with the p values.

Meta-analyses encompassing over 46,000 hip arthroplasties have highlighted several advantages of the DAA in the geriatric population [8]. The DAA utilizes an internervous, intermuscular plane (between the tensor fasciae latae and sartorius muscles), thereby sparing the posterior soft tissues and preserving abductor function. Compared to the PA, the DAA is associated with significantly lower blood transfusion rates (6.62% vs. 14.52%, p < 0.005), less damage to the gluteus minimus muscle as observed on postoperative MRI (36.84% vs. 65.79%, p < 0.005) and a significantly lower dislocation rate (0.84% vs. 1.82%, p < 0.001) [8]. Figure 4 illustrates these perioperative differences. These benefits likely translate into quicker early rehabilitation as DAA patients often report less pain and earlier return of functional gait in the initial weeks post-surgery. A separate meta-analysis of over 44,000 patients corroborated the short-term advantages of DAA, including shorter hospital stay (p = 0.01) and reduced incision length (p = 0.001), while demonstrating comparable rates of all-cause revision (p = 0.40), intraoperative fracture and periprosthetic fracture (p = 0.11) between the two approaches [9].

## 8. Mid-term Parity

While the DAA offers faster subjective recovery in the early postoperative weeks, mid-term outcomes (≥5 years) between DAA and PA show parity [9]. Both approaches achieve comparable patient-reported outcome scores, with no significant difference in Harris Hip Score between cohorts [9]. Importantly, however, the DAA maintains a significantly lower risk of postoperative hip dislocation compared to the PA (approximately 0.84% vs. 1.82% in large series, *p* < 0.001) [8]. This reduced dislocation risk is often attributed to the preservation of posterior soft-tissue structures with the DAA, which adds a measure of intrinsic stability to the hip. Overall, the choice of approach should be individualized, but current evidence supports the DAA as a safe option that confers early benefits without compromising mid-term results in the geriatric population.

## 9. Risk Stratification and Complication Mitigation

Beyond mechanical failure, periprosthetic joint infection (PJI) and patient comorbidities represent primary challenges to long-term success in geriatric THA [10].

## 10. Periprosthetic Joint Infection (PJI)

PJI remains a devastating complication, with a cumulative incidence of 1.44% at 15 years following primary THA in a population-based cohort of over 100,000 patients [10]. Notably, 62% of infections occurred within 2 years of surgery and 98% within 10 years, highlighting the importance of both early and long-term surveillance [10]. While early total hip arthroplasty failures were often attributed to polyethylene wear, advances in material technology have reduced wear-related revisions, and contemporary analyses identify periprosthetic joint infection as one of the leading causes of revision surgery [11].

### Surgical Factors:

Prolonged operative time (≥120 minutes) is associated with increased infection risk, as is extended hospital length of stay [12–13]. Operative times exceeding 90 minutes confer a 1.6-fold increased risk compared to procedures under 60 minutes [14].

### Patient Factors:

Morbid Obesity (BMI ≥40 kg/m^2^) is associated with substantially increased risk of periprosthetic joint infection after THA [15]. Diabetes mellitus increases the risk of periprosthetic joint infection after total joint arthroplasty, with affected patients demonstrating higher odds of infection than non-diabetic patients [16].

#### Management Strategies:

Beyond addressing modifiable risk factors, many arthroplasty centers have adopted standardized perioperative pathways (including ERAS/outpatient protocols) to reduce variation in care and improve recovery after THA [17].

In addition, International Consensus Meeting (ICM)–aligned practices frequently include preoperative *Staphylococcus aureus* screening with targeted decolonization using intranasal mupirocin and chlorhexidine bathing, which has been associated with lower surgical site infection and periprosthetic joint infection rates in total joint arthroplasty populations [18].

Because malnutrition and hypoalbuminemia are independently associated with increased postoperative infection risk, contemporary perioperative pathways also emphasize preoperative nutritional assessment and optimization in elderly patients undergoing total hip arthroplasty [19].

#### Glycemic Control:

Poor preoperative glycemic control has been identified as a significant risk factor for periprosthetic joint infection following total hip and knee arthroplasty, with patients with diabetes and perioperative hyperglycemia demonstrating higher postoperative infection rates compared with normoglycemic patients [20].

Accordingly, contemporary perioperative optimization pathways recommend routine assessment of glycemic status and targeted preoperative glucose control as part of infection risk mitigation strategies prior to elective total hip arthroplasty [21].

## 11. The Role of Emerging Technology: AI and 3D Printing

The integration of robotic assistance and artificial intelligence (AI) into total hip arthroplasty has enabled real-time intraoperative assessment of component positioning, with AI-enhanced navigation systems providing fluoroscopy-based feedback on cup inclination and anteversion to improve the accuracy and reproducibility of implant alignment [22].

In addition, three-dimensional (3D) printed patient-specific anatomical models are increasingly employed in complex primary and revision THA to facilitate preoperative planning, optimize implant selection, and restore the native hip center of rotation [23].

Together, these technological adjuncts support a more individualized approach to reconstruction and may augment surgical decision-making in geriatric patients with compromised bone quality or complex anatomy.

## 12. Discussion

Contemporary evidence supports a multifaceted approach to geriatric total hip arthroplasty that integrates fixation strategy, surgical approach, and surgeon expertise. In patients over 75 years of age with osteoporotic bone, cemented femoral fixation remains a strongly supported strategy for achieving early implant stability, facilitating immediate weight-bearing, and reducing the risk of intraoperative periprosthetic fracture, as demonstrated by superior rotational stability in biomechanical fracture models [6].

These mechanical advantages may be further complemented by minimally invasive approaches such as the Direct Anterior Approach, which has been associated with shorter hospital length of stay and improved early functional recovery when implemented within Enhanced Recovery After Surgery (ERAS) protocols [8].

At the same time, the increasing predominance of cementless techniques and digital workflows in contemporary practice has raised concerns regarding declining exposure to cementation techniques during surgical training, despite ongoing evidence supporting their value in elderly patients [3]. Ensuring that future orthopedic surgeons maintain proficiency in both modern computer-assisted technologies and foundational fixation techniques will be critical as the volume of geriatric arthroplasty and fracture care continues to expand.

## 13. Major Key Points

1. **Mechanical Stability:** Cemented femoral stems provide significantly higher rotational stability compared to cementless designs in fracture models.
2. **Perioperative Advantage:** Geriatric patients undergoing cemented THA have, on average, shorter hospital stays and higher home-discharge rates than those with cementless stems.
3. **Surgical Approach Trends:** The Direct Anterior Approach (DAA) is associated with reduced muscle damage and lower blood transfusion rates as well as a lower dislocation rate, compared to the Posterior Approach.
4. **PJI Risk Factors:** Obesity and diabetes are major predictors of periprosthetic joint infection, and longer operative times significantly increase infection risk. Comprehensive risk optimization and strict adherence to infection prevention protocols are critical in elderly THA patients.
5. **Technological Integration:** AI-enhanced robotics and 3D-printed anatomical models are improving surgical accuracy and helping personalize reconstruction strategies in complex geriatric cases.
6. **Long-term Durability:** Despite early differences in fixation, both cemented and cementless THA demonstrate excellent 10-year survivorship in older patients. This parity in long-term outcomes underscores that the fixation method should be tailored to patient bone quality and surgeon expertise, as both can achieve durable results when applied appropriately.

## 14. Outstanding Questions

### Training and Skills:

How will the decline in cementation experience among new surgeons impact complication rates as the geriatric THA volume grows? Can orthopedic training programs adapt to ensure proficiency in cementing techniques despite the predominance of cementless implants in younger patients?

### Augmented Planning:

Can AI-driven preoperative planning and intraoperative guidance eventually eliminate the fracture-risk gap between cemented and cementless fixation in osteoporotic bone?

### Biofilm and Microbiome:

What is the long-term impact of the periprosthetic “joint microbiome” and systemic dysbiosis on the incidence of late-onset PJI in THA? Could modulation of microbiota become part of infection prevention in the future?

### Cost-Benefit of Technology:

Will the cost–benefit ratio of robotic-assisted THA be conclusively proven for routine primary geriatric cases, or will such technology remain reserved for complex reconstructions? The answer may depend on future improvements in efficiency and reductions in system costs.
